# Robust noninvasive detection of hyperglycemia in mouse models of metabolic dysregulation using the novel Urination Index biomarker

**DOI:** 10.1038/s41684-025-01648-8

**Published:** 2025-11-21

**Authors:** Sebastian Brachs, Morten Dall, Leonie-Kim Zimbalski, Yohan Santin, Christian Oeing, Knut Mai, Angelo Parini, Stefano Gaburro, Thomas Svava Nielsen

**Affiliations:** 1 Department of Endocrinology and Metabolism, European Reference Network on Rare Endocrine Diseases, Charité – Universitätsmedizin Berlin, corporate member of Freie Universität Berlin and Humboldt–Universität zu Berlin, Berlin, Germany; 2 German Centre for Cardiovascular Research (DZHK), Berlin, Germany; 3 Novo Nordisk Foundation Center for Basic Metabolic Research, University of Copenhagen, Copenhagen, Denmark; 4 IHU HeathAge, Institute of Metabolic and Cardiovascular Diseases INSERM UMR 1297, Université Toulouse Paul Sabatier, Toulouse, France; 5 Deutsches Herzzentrum der Charité (DHZC), Department of Cardiology, Angiology and Intensive Care Medicine, Charité – Universitätsmedizin Berlin, corporate member of Freie Universität Berlin and Humboldt–Universität zu Berlin, Berlin, Germany; 6 Department of Human Nutrition, German Institute of Human Nutrition (DIfE) Potsdam–Rehbruecke, Nuthetal, Germany; 7 NutriAct–Competence Cluster Nutrition Research Berlin–Potsdam, Nuthetal, Germany; 8 German Center for Diabetes Research (DZD e.V.), Neuherberg, Germany; 9 Digilab Solutions, Tecniplast S.p.A., Maggio, Italy

**Keywords:** Diabetes, Diagnostic markers, Kidney, Metabolism, Kidney diseases

## Abstract

Blood glucose is one of the most essential parameters in metabolic research. Yet, accurate blood glucose monitoring in mouse models of diabetes is challenging owing to the substantial stress associated with the measurements and the variability in diabetes development among experimental mouse models. This variability requires frequent blood glucose measurements, which provide only intermittent data and may not accurately reflect continuous metabolic changes. Here, to address these issues, we have utilized the Tecniplast DVC system to monitor bedding moisture, enabling the detection of increased urination (polyuria) in mice, a primary symptom of diabetes. Polyuria is a hallmark of (undiagnosed/untreated) diabetes, and we revealed high correlations between bedding moisture and blood glucose during hyperglycemia. Thus, our developed algorithm enhances animal welfare by reducing the need for invasive blood glucose tests and enabling noninvasive, continuous assessment of hyperglycemia onset, progression and severity directly within the mice’s home cage. The continuous monitoring of polyuria allows the detailed analysis of temporal and circadian urination patterns and enables assessment of the efficacy of glucose-lowering interventions, which is critical in developing new pharmacological treatments. We propose that this innovative approach of a novel digital biomarker, the Urination Index, offers a substantial advance in the methodology for diabetes research in mouse models, improves animal welfare by reducing the need for invasive blood glucose tests and enhances the reliability of data and the quality of life for the animals involved.

## Abstract (web summary)

This Article presents a new digital biomarker of diabetes, the Urination Index, to monitor bedding moisture in mice. This noninvasive method reduces the need for invasive blood glucose tests, improving animal welfare and data reliability.

## Main

Diabetes mellitus (DM) is a major global health problem affecting more than 800 million people and causing severe challenges for healthcare systems^1^. Chronic hyperglycemia (blood glucose >10 mM) is a critical factor^2^, and is related to 43% of all deaths of patients under 70 years old with DM, cardiovascular and other diseases^3^. To address this global burden, research relies on mouse models that mimic hyperglycemia, insulin resistance or DM, but these come with substantial limitations complicating their translation. Blood glucose assessment is indispensable in the animal models used in metabolic research and pharmacology, but it is invasive with known drawbacks^4^. Various mouse models are utilized in metabolic and diabetes research to develop insulin resistance or insulin loss. The non-obese diabetic (NOD) mouse strain exhibits spontaneous autoimmune destruction of pancreatic β cells and is the most common genetic model of type 1 DM (T1DM), which is characterized by hypoinsulinemia. Alternatively, rapid loss of endogenous insulin can be achieved by treatment of wild-type (WT) mice with the β cell-specific cytotoxins streptozotocin (STZ) or alloxan, providing a T1DM-like phenotype on any genetic background. The severely obese ob/ob and db/db mouse strains, lacking the hormone leptin or the leptin receptor, respectively, are the primary genetic models of type 2 DM (T2DM), characterized by moderate to severe insulin resistance. However, the most widely used model of metabolic dysfunction is the diet-induced obese (DIO) C57BL/6 mouse, representing a prediabetic state of glucose intolerance and mild insulin resistance^5–9^. Commonly for all these models, the onset of hyperglycemia is difficult to detect as blood glucose is typically captured via a single measurement, providing only a snapshot. Moreover, the measurements are mostly done during the light phase when mice rest, missing the postprandial phase, or via a glucose tolerance test, which is even more invasive^10^. Therefore, accurate monitoring of blood glucose is a major challenge, primarily owing to the sampling stress triggering hyperglycemia and the inherent variability in disease progression across and within models^4,11–15^. Traditional sampling using glucometers is invasive and labor intensive and offers only intermittent glucose snapshots that fail to capture dynamic fluctuations^16^. Continuous glucose monitoring (CGM) devices are cost intensive and require specialized surgical skills and anesthesia, which have important glucose regulating effects^17–19^. Thus, reliable glucose monitoring is difficult in mice and the necessary procedures to retrieve the measurements may affect experimental outcomes^20,21^.

Excessive urination, known as polyuria, is one of the first clinical manifestation and common symptom of hyperglycemia in undiagnosed or poorly managed DM accompanied by polydipsia and polyphagia^22^. Voiding behavior, the pattern and frequency of urination, is regulated by a circadian rhythm and is known to be altered in DM^23–25^. The most common methods to analyze urine and voiding behavior in laboratory animals are void spot assays (VSAs), catheterization and metabolic cages, which are all associated with stress^26–31^. VSAs, commonly used to assess urination in rodents^29,32^, require removal from the home cage and can cause substantial stress, potentially skewing research findings. Furthermore, VSAs merely provide a time-limited, temporary diagnostic snapshot.

As hyperglycemia has a complex diurnal rhythm and is a main driver of polyuria, we hypothesized that monitoring of urination patterns could provide valuable insights about blood glucose levels in mouse models of DM. Therefore, we implemented an innovative approach using changes in bedding moisture as a measure of polyuria for noninvasive continuous monitoring of hyperglycemia. To assess bedding moisture, we utilized the Digital Ventilated Cage (DVC; Tecniplast) sensing technology. The DVC system automatically records home cage data for multiple parameters for daily routine tasks in the animal husbandry management and animal welfare support^33,34^. The DVC technology tracks the cages using 12 capacitive sensors embedded under each cage to detect changes in electromagnetic field strength, enabling continuous, noninvasive monitoring of parameters such as movement and bedding moisture. Multiple approaches have already employed the DVC system to derive further parameters, such as cage aggression, behavioral traits, activity or circadian profiles^35–40^.

In this work, we assessed increased cage urination (polyuria) in T1DM and T2DM mouse models by detecting bedding moisture using the DVC system and were able to correlate it with polydipsia, polyphagia and hyperglycemia. As a T1DM model, we treated C57BL/6J female mice with STZ to induce β cell loss, followed by insulin deficiency, hyperglycemia, polydipsia and polyuria, all typical characteristics of human T1DM^41^. For hyperglycemia and T2DM, we employed the ob/ob mouse model, which develops hyperphagia, obesity, hyperglycemia and insulin resistance, mimicking several key features of human T2DM^42^. We developed a novel digital biomarker, the Urination Index (UI), for the noninvasive assessment of urination and designed the UrinatoR app for its analysis (https://tsnscientific.com/urinator hosted at: https://cbmr-rmpp.shinyapps.io/UrinatoR/). This approach enables sample-free continuous tracking of the progression of hyperglycemia within the home cage. Our innovative approach adheres to the 3Rs principle by reducing invasive sampling, handling and, therefore, stress, improving animal welfare and increasing data reliability. Furthermore, unlike intermittent measurements, longitudinal and circadian urination patterns can be identified in high temporal resolution, including during the dark phase when mice are active. Finally, we could reverse the polyuria phenotype by pharmacological blood glucose-lowering intervention in hyperglycemic STZ-treated mice. This real-time remote monitoring of disease onset, progression and severity in the home cage environment enables timing of interventions or treatments according to the onset of hyperglycemia, limiting model variability and improving outcomes of metabolic, diabetic and pharmacological research.

## Results

### Basic urination algorithm

**Fig. 1:**
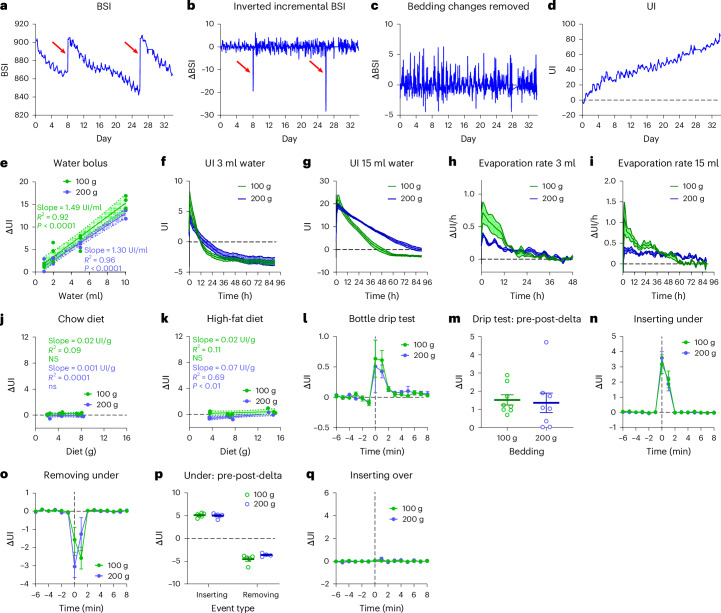
Derivation of the UI algorithm and in vitro validation. **a**, The DVC BSI from one cage. Two bedding changes are indicated (arrows). **b**, The conversion of the BSI to incremental changes and inversion of the sign. **c**, Incremental changes after the removal of bedding changes. **d**, The UI generated by the accumulation of processed incremental data. **e**, A dose-response test of injecting water in cages without mice: 1 ml, 2 ml, 5 ml and 10 ml were injected in cages with 100 g or 200 g of bedding (*n* = 3). The 95% CI (shaded area) and regression results are indicated (ANCOVA with covariate water bolus and factor bedding amount: 100 g: *R*² = 0.92, *P* < 0.0001; 200 g: *R*² = 0.96, *P* < 0.0001). **f**,**g**, The UI change during evaporation of 3 ml (**f**) or 15 ml (**g**) of water from bedding (mean ± s.e.m., *n* = 3). **h**,**i**, The rate of evaporation of 3 ml (**h**) and 15 ml (**i**) of water, calculated as the slope of **f** and **g** (mean ± s.e.m., *n* = 3). **j**,**k**, The UI response and regression results to adding 1, 2 and 4 chow diet pellets (*n* = 3, linear regression: 100 g: *R*² = 0.09, *P* = NS; 200 g: *R*² = 0.0001, *P* = NS) (**j**) or high-fat diet pellets (*n* = 3, linear regression: 100 g: *R*² = 0.11, *P* n.s.; 200 g: *R*² = 0.69, *P* < 0.01) (**k**). **l**, The UI response to bottle dripping during cage handling (mean ± s.e.m., *n* = 8). **m**, The total change in UI after a cage handling event (mean ± s.e.m., *n* = 8). **n**, The UI response to insertion of a cage in the slot under the monitored cage (mean ± s.e.m., *n* = 6). **o**, The UI response to the removal of a cage from the slot under the monitored cage (mean ± s.e.m., 100 g: *n* = 5; 200 g: *n* = 4). **p**, The total change in UI when a cage is inserted or removed under the monitored cage (mean ± s.e.m., Inserting: *n* = 6; removing: 100 g: *n* = 5; 200 g: *n* = 4). **q**, The UI response to the insertion of a cage in the slot above the monitored cage (mean ± s.e.m., *n* = 3). Source data

The DVC detects increasing bedding moisture as a decline in electromagnetic field strength and records this as the Bedding Status Index (BSI, Fig. 1a). To obtain a metric that increases with added moisture, the BSI was converted into inverse incremental changes for each data point (Fig. 1b). After removal of signal corresponding to bedding changes (Fig. 1c), the UI was calculated as the cumulative development of inverted and processed BSI (Fig. 1d). Hence, UI is a cumulative metric, the inverse of the BSI with the discontinuities of bedding changes removed, having the same scale and arbitrary unit as the BSI.

### In vitro tests of water, diet and cage-handling effects

To establish the relationship between the UI and added water or diet in cages, we conducted controlled ‘in vitro’ experiments without animals, using cages with varying amounts of bedding. The UI signal showed a strong linear relationship with the volume of added water (Fig. 1e). Since slopes were not dependent on bedding amount (analysis of covariance (ANCOVA), *P* = not significant (NS)), we could estimate the average effect of water on the signal to be 1.4 UI/ml. Conversely, evaporation rates were dependent on bedding amount. With 3 ml and 15 ml volume injected, the UI signal had a steeper initial decline and reached a plateau sooner in cages with 100 g than with 200 g bedding (Fig. 1f-g). Calculating the evaporation rate revealed that irrespective of initial water content, 200 g bedding promoted a more constant evaporation rate than 100 g, indicating that more bedding stabilized the UI signal (Fig. 1h-i). Unlike water, chow or high-fat diet (HFD) pellets had minimal effects on the UI signal (0.001-0.07 UI/g, that is, <5% of an equivalent mass of water, Fig. 1j-k), suggesting that diet spillage or shredding is unlikely to contribute to the estimate of urination. Since there is a risk of bottle spillage when handling cages, we simulated a standard cage-handling procedure where cages were removed, opened, closed, and returned to the DVC (Fig. 1l). Indeed, dripping can cause a substantial UI change, with an overall average of 1.4 ± 0.3 UI units in both bedding groups (Fig. 1m). However, the inter-cage differences varied considerably between 0.02-4.70 UI units (corresponding to 0.01-3.40 ml). As the DVC electrodes are omnidirectional, they are also expected to detect the proximity of water in a bottle immediately below. Consequently, we inserted and removed surrounding cages and indeed, inserting a cage with a full bottle below caused a substantial UI increase in the cage above (Fig. 1n). Vice versa, a UI reduction was observed in the upper cage when removing that below (Fig. 1o). The insertion/removal effect below was equal and opposite to that of the cage above, both causing an offset of 5 UI units (Fig. 1p). As expected, inserting/removing a cage above did not affect the UI of the cage below (Fig. 1q). Altogether, our tests indicate that UI is a reliable and selective measure of changes in bedding fluid content, but also that exclusion of measurements associated with cage-handling and insertion/removal events is critical in data processing.

### UI scales with increasing housing density

**Fig. 2:**
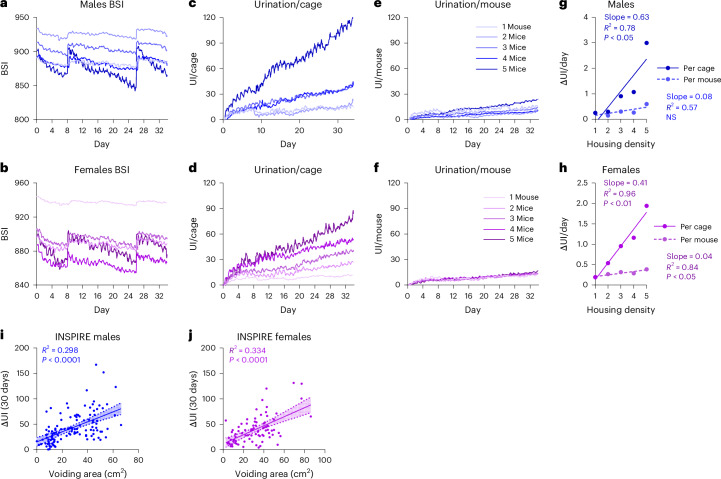
UI assessment according to housing density and in the INSPIRE cohort. **a**,**b**, The BSI from five cages per sex with 1, 2, 3, 4 or 5 male (**a**) or female (**b**) CD1 mice (one cage per sex for each housing density condition). **c**,**d**, The UI for each cage of male (**c**) and female (**d**) mice. **e**,**f**, The UI normalized for housing density of each cage of male (**e**) and female (**f**) mice. **g, h**, The relationship between UI and housing density in male (**g**) and female (**h**) mice before (linear regression: males: *R*² = 0.78, *P* < 0.05; 200 g: *R*² = 0.0001, *P* = NS) and after normalization. **i**,**j**, The correlation and 95% CI (shaded area) between the 30-day UI increase and the total urine spot area during VSA in male (*n* = 123 cages, linear regression: *R*² = 0.298, *P* < 0.0001, **i**) and female (*n* = 87 cages, linear regression: *R*² = 0.334, *P* < 0.0001, **j**) outbred SWISS mice from the INSPIRE cohort. Source data

To evaluate our approach, we analyzed the linear scalability of the UI by performing a housing density test with increasing numbers of male and female CD1 mice per cage (Fig. 2a-b). BSI was converted to UI per cage, with cage density of 1, 2, 3, 4, or 5 mice (Fig. 2c-d). Thereafter, we normalized by housing density to derive UI per mouse (Fig. 2e-f). The urination rate per cage calculated as the slope of the UI curve showed a strong linear correlation with cage density in both sexes (linear regression: males: 0.63 UI/mouse/day, *R*^2^ = 0.78, *P* < 0.05, Fig. 2g; females: 0.41 UI/mouse/day, *R*^2^ = 0.96, *P* < 0.01, Fig. 2h). After housing density normalization, this correlation was no longer present for males (Fig. 2g). Although a slight correlation was still present after normalization in females (0.04 UI/mouse/day, *R*^2^ = 0.84, *P* < 0.05, Fig. 2h), these results demonstrate the linear scalability and robustness of our approach across different housing densities.

### UI correlates with voiding behavior

We processed DVC data of male and female mice from the INSPIRE cohort^40,43^, in which SWISS outbred mice were longitudinally phenotyped to investigate aging. These data were used to correlate the UI to results of the voiding behavior test. Here, we found a positive correlation between total voiding spot area and UI indicating that the UI is a valid measure of urinary output (linear regression: males: *R*² = 0.55, *P* < 0.0001 (Fig. 2i); females: *R*² = 0.58, *P* < 0.0001 (Fig. 2j)).

### Ob/ob mice show increased UI

**Fig. 3:**
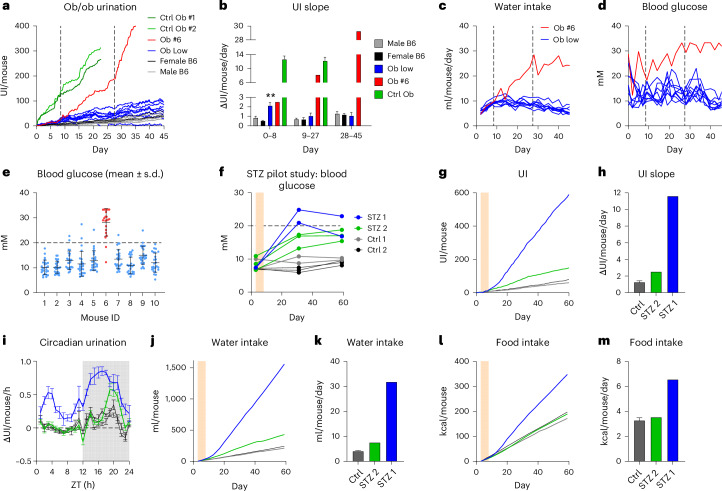
Diabetes pilot studies. **a**, Urination of ob/ob mice: UI per mouse for nonresponder ob/ob (Ob low, *n* = 9 cages, individually housed), responder ob/ob mouse (Ob #6, *n* = 1), positive control ob/ob (Ctrl Ob #1, Ctrl Ob #2, *n* = 2 cages, individually housed), female C57BL/6JRj (female B6, *n* = 4 cages, each with 4 mice) and male C57BL/6JRj (male B6, *n* = 4 cages, each with 4 mice). The dashed lines indicate the time of response changes in Ob #6. **b**, The average daily urination rate in each study period (mean ± s.e.m., group-wise ANOVA, **P < 0.01 compared to male B6. **c**,**d**, Time course of individual daily water intake (**c**) and blood glucose (**d**) in Ob low mice and Ob #6. **e**, Blood glucose levels for each mouse in Ob low (blue) and Ob #6 (red) (mean ± s.d.). **f**, Blood glucose time course for each C57BL/6J mouse in the STZ pilot study (mice from STZ-treated cages: STZ 1: blue (*n* = 2 mice) and STZ 2: green (*n* = 3), mice from control cages: Ctrl 1: gray (*n* = 2) and Ctrl 2: black (*n* = 2)). STZ was dosed on days 3–8 (shaded area). **g**, UI normalized for housing density. **h**, Average daily urination rate (UI slope days 10–60, Ctrl: mean ± s.e.m., *n* = 2 cages; STZ 1/2: *n* = 1 cage). **i**, Circadian urination patterns (days 10–60, individual cages, Ctrl: mean ± s.e.m., *n* = 2 cages and STZ per cage, with 2 (STZ 1: blue; Ctrl 1: gray) and 3 (STZ 2: green; Ctrl 2: black) mice). Time of circadian urination patterns is depicted as Zeitgeber time (ZT). **j**, Cumulative water intake. **k**, Average daily water intake (water intake slope days 10–60, Ctrl: mean ± s.e.m., *n* = 2). **l**, Cumulative food intake. **m**, Average daily food intake (food intake slope days 10–60, Ctrl: mean ± s.e.m., *n* = 2 cages). Source data

As initial proof of concept, we monitored two severely hyperglycemic ob/ob male mice (labeled control (Ctrl) Ob #1 and Ctrl Ob #2), with constant blood glucose >33.3 mM. Both mice showed dramatically increased UI compared to WT C57BL/6J male and female controls (Fig. 3a). Consequently, we conducted another study with ten individually housed ob/ob males, monitored from 3 weeks of age to study UI progression during hyperglycemia development. Compared to WT, nine ob/ob mice had only mildly elevated UI (Ob low; Fig. 3a). However, one ob/ob mouse (Ob #6) showed severe polyuria, starting from day 9 (Fig. 3a). On day 28, it also developed a stereotypic behavior of excessive grooming on the water nozzle, causing spillage and further exacerbated the increase in UI. By calculating the slope of UI curves, we derived the average daily ΔUI for each phase of the study (Fig. 3b). In the first 8 days, the ΔUI of the nine Ob low mice was 2.5-fold elevated (Ob low mice: 2.2 ± 0.3 UI/mouse/day) compared to male WT mice (0.9 ± 0.1 UI/mouse/day, two-way ANOVA, *P* < 0.01). After day 9, however, their ΔUI decreased by ~50% and no longer differed from WT. In contrast, while Ob #6 was comparable to Ob low in the initial phase (2.5 ΔUI/mouse/day), ΔUI increased 11.5-fold compared to WT between day 9 and 27 (8.3 ΔUI/mouse/day), where it was comparable to ΔUI of Ctrl Ob (12.3 ± 1.0 ΔUI/mouse/day). After day 28, the ΔUI of Ob #6 further increased 23-fold over WT; however, this was co-caused by water spillage through the stereotypic grooming behavior.

The water intake mirrored the UI results, with Ob low exhibiting an initial increase (day 2: 5.8 ± 0.2 ml/mouse/day, peak day 14: 10.4 ± 0.4 ml/mouse/day), followed by a gradual decrease (day 44: 6.1 ± 0.4 ml/mouse/day; Fig. 3c). Consistently, Ob #6 diverged from Ob low and water intake progressively increased between days 9 and 28 (day 9: 10.1 ml/mouse/day), until stabilizing after day 28 (between 24 and 29 ml/mouse/day). Correspondingly, blood glucose analysis revealed that Ob #6 was the only mouse that developed severe hyperglycemia and the polyuria onset coincided with the point at which blood glucose consistently exceeded 20 mM (Fig. 3d), suggesting a threshold effect. While Ob low occasionally had blood glucose levels approaching or exceeding 20 mM, most measurements showed only mild to moderate hyperglycemia (range: 10.1 ± 2.9 mM Ob #1 to 15.0 ± 3.6 mM Ob #9; Fig. 3d,e).

### STZ pilot study

To explore the generalizability of the UI, we employed data of a STZ-induced T1DM pilot with 10 C57BL/6J female WT mice: 5 mice receiving STZ and 5 vehicle-only Ctrl treatment. Per treatment, groups were housed in two cages with two and three mice. In week 4, the blood glucose response was strongest in both mice of cage labeled STZ 1 (22.9 ± 2.0 mM), while the response in STZ 2 was only mild (15.8 ± 1.3 mM; Fig. 3f). Interestingly, the relatively small difference of mean blood glucose between the two STZ cages was associated with a striking difference in UI (Fig. 3g). Compared to Ctrl (1.3 ± 0.2 UI/mouse/day), UI was increased twofold in STZ 2 (2.5 UI/mouse/day), but ninefold in STZ 1 (11.6 UI/mouse/day; Fig. 3h). Analysis of circadian urination revealed that polyuria in STZ 1 occurred throughout the 24-h cycle, peaking in the middle of the dark phase, where feeding-induced hyperglycemia would be expected to amplify diuresis in severely hyperglycemic animals (Fig. 3i). Likewise, the UI of STZ 2 peaked in the late dark phase but was virtually identical to WT during the rest of the circadian cycle, suggesting that these mice only become severely hyperglycemic postprandially. Again, the water intake mirrored the UI (Fig. 3j), with an increase of 1.9-fold in STZ 2 (7.6 ml/mouse/day) and 7.8-fold in STZ 1 (31.8 ml/mouse/day) compared to WT (4.1 ± 0.3 ml/mouse/day; Fig. 3k). Remarkably, food intake in STZ 1 was increased twofold (6.6 kcal/mouse/day), while STZ 2 (3.5 kcal/mouse/day) was virtually identical to WT (3.3 ± 0.2 kcal/mouse/day; Fig. 3l,m). Considering the relatively modest blood glucose difference between the two STZ cages, the disproportional potentiation of the three key symptoms of DM—polyuria, polydipsia and polyphagia—seems extraordinary and reinforces the notion of a threshold effect occurring when blood glucose exceeds 20 mM.

### STZ main study

**Fig. 4:**
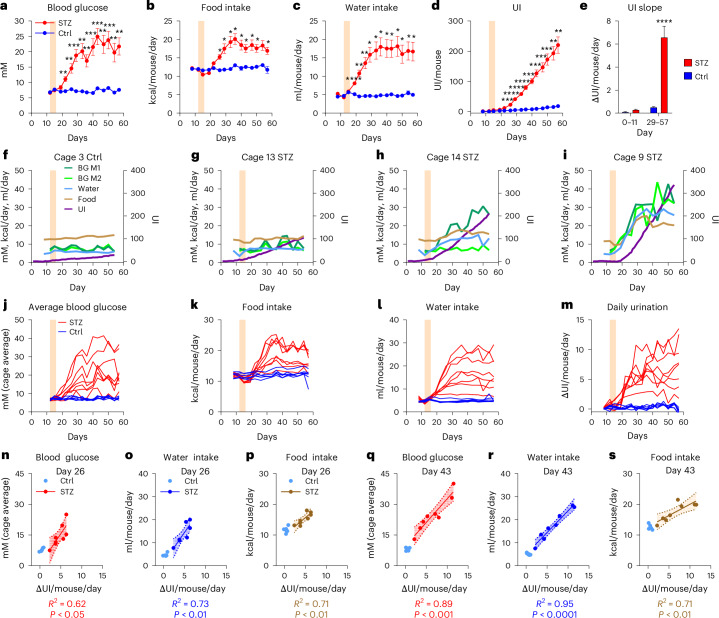
STZ main study: primary effects and correlations. **a**–**d**, Time course development of blood glucose (**a**), food intake (**b**), water intake (**c**) and UI (**d**) following STZ dosing (shaded area) in control cages (*n* = 6) and STZ-treated cages (*n* = 8) of pair-housed female C57BL/6J mice (mean ± s.e.m., Ctrl: *n* = 12 mice; STZ: *n* = 16 mice, tested with repeated-measures two-way ANOVA adjusted with Tukey’s multiple comparisons test). **e**, The average daily UI before (day 0–11) and after STZ (day 29–57) (linear regression slope ± s.e.m. of **d**, ANCOVA with covariate treatment and factor time). **f**–**i**, Individual time course data from representative cages of control (M1: mouse-1, M2 mouse-2, **f**), low-responder STZ (**g**), single-responder STZ (**h**) and dual high-responder STZ (**i**). Blood glucose (BG) levels are from individual mice, while food intake, water intake and UI are normalized for housing density. Blood glucose levels (mM), food intake (kcal/mouse/day) and water intake (ml/mouse/day) are on the same scale and thus plotted together on the left axis, but with different units. UI is plotted on the right axis. **j**–**m**, Time course data for individual cages of control (blue) and STZ (red), including blood glucose (cage average) (**j**), daily food intake (**k**), daily water intake (**l**) and daily UI change (**m**). **n**–**p**, Correlations (linear regression) and 95% CI (shaded area) between daily UI change and blood glucose (cage average) (**n**), water intake (**o**) and food intake (**p**) in the STZ group 10 days after treatment. **q**–**s**, Correlations (linear regression) and 95% CI (shaded area) between daily UI change and blood glucose (cage average) (**q**), water intake (**r**) and food intake (**s**) in the STZ group 27 days after treatment. *P < 0.05, **P < 0.01, ***P < 0.001 and ****P < 0.0001. Source data

Encouraged by the previous observations, we performed a follow-up study with 12 Ctrl and 16 STZ-treated C57BL/6J WT females and monitored them 8 weeks. They were separated by treatment and pair housed in the DVC. Individual blood glucose levels (Fig. 4a) as well as cage-based food (Fig. 4b) and water intake (Fig. 4c) were assessed twice weekly. The UI was tracked continuously and was cage based (Fig. 4d). Before treatment, the UI was low and similar between cages; thereafter its slope increased 12.6-fold in STZ cages compared to Ctrl cages (6.6 ± 1.0 UI/mouse/day versus 0.5 ± 0.05 UI/mouse/day, two-way ANOVA, *P* < 0.0001; Fig. 4e). On inspection of the combined data for each cage, a dose-dependent effect of hyperglycemia on the other parameters was immediately apparent. In Ctrl cages, where blood glucose remained stable, UI, food and water intake were also constant (Fig. 4f and Supplementary Fig. 1a–f). Conversely, the magnitude of the changes in food, water and UI essentially reflected the average magnitude of the STZ-mediated hyperglycemia of both mice in a STZ cage (Fig. 4g–i and Supplementary Fig. 1g–n). Importantly, polyuria was clearly detectable, even if only one mouse in a cage developed hyperglycemia, indicating that the UI is also reliable for detection of individual cases of polyuria in group-housed mice (Fig. 4h). Given the variable response to STZ, a wide range of values was observed for blood glucose, food and drink intake and daily urination measured as average per cage. (Fig. 4j–m). Consequently, we investigated the diagnostic potential of UI as a quantitative indicator of hyperglycemia, polydipsia and polyphagia.

Average blood glucose per cage positively correlated with UI already on day 22, 6 days post-STZ (Supplementary Fig. 2a). On day 26, we found a strong positive correlations between UI and blood glucose, water and food intake (Fig. 4n–p), which even increased by day 43 (Fig. 4q–s). In fact, the linear relationship between UI and blood glucose, water and food intake remained strong from day 26 until the end, except for day 33 (Supplementary Fig. 2a–c). The polyphagia, observed in STZ #1 in the pilot, was recapitulated in 50% of STZ cages (Supplementary Fig. 3a). These mice also showed the highest increase in blood glucose (Supplementary Fig. 3b), UI (Supplementary Fig. 3c) and water intake (Supplementary Fig. 3d) compared to the Ctrl cages. Only food intake exhibited this seemingly dichotomous response pattern.

### Subdivision of the STZ main study mice into STZ high and STZ low responders

**Fig. 5:**
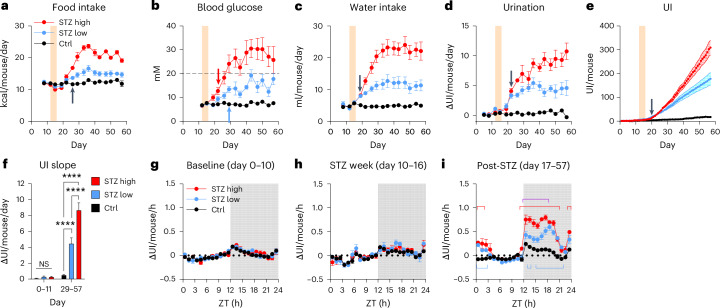
STZ main study: high/low responder analysis. **a**–**e**, Time course development (mean ± s.e.m.) in ‘STZ high’ responder (*n* = 4) and ‘STZ low’ responder (*n* = 4), and Ctrl cages (*n* = 6) of food intake (**a**), blood glucose (**b**), water intake (**c**), daily UI change (**d**) and UI (**e**) following STZ dosing (shaded area). The main effects and the interaction between groups and time were highly significant for all of **a**–**e** tested by repeated-measures two-way ANOVA adjusted with Tukey’s multiple comparisons test. The arrows denote the first significant divergence of ‘STZ high’ or ‘STZ low’ groups from Ctrl (*P* < 0.05). The black arrows indicate simultaneous divergence of ‘STZ high’ and ‘STZ low’ from Ctrl, while the divergence at different time points is indicated in arrows matching the group color. All other indicators of significance have been omitted for clarity. **f**, The average daily urination rate before (days 0–11) and after STZ (days 29–57) (mean ± s.e.m., group-wise ANOVA, *P* < 0.0001). **g**–**i**, The circadian change in UI (mean ± s.e.m.) before STZ (**g**), during STZ treatment (**h**) and after STZ (**i**). Significant differences from Ctrl for each STZ group are indicated with brackets in the group color, and purple bracket indicates differences between ‘STZ high’ and ‘STZ low’ (mean ± s.e.m., tested by repeated-measures two-way ANOVA adjusted with Tukey’s multiple comparisons test, *P* < 0.05). *P < 0.05, **P < 0.01, ***P < 0.001 and ****P < 0.0001. Source data

Considering the differing response to STZ, we grouped the STZ cages into low responder (‘STZ low’) and high responder (‘STZ high’) according to whether the resulting polyphagia was mild or severe, respectively. While food intake increased by ~20% in ‘STZ low’, polyphagia was indeed severe in ‘STZ high’, peaking at about twice that of the Ctrl, with both STZ groups becoming significantly different from control at day 26 (two-way ANOVA, *P* < 0.05; Fig. 5a). Again, the main driver seems to be whether blood glucose exceeds 20 mM as this was a consistent differentiator between groups after day 26 (Fig. 5b). This observation supports a tipping point existence at 20 mM. Although similar trajectories were found for glucose (two-way ANOVA, *P* < 0.001; Fig. 5b), daily water intake (two-way ANOVA, *P* < 0.0001; Fig. 5c) and urination (two-way ANOVA, *P* < 0.0001; Fig. 5d), the temporal dynamics differed. Glucose was elevated from day 22 in ‘STZ high’ (Fig. 5b, red arrow) and from day 29 in ‘STZ low’ (Fig. 5b, blue arrow) compared to Ctrl. In contrast, polydipsia emerged significantly in both groups from day 19, only 3 days post-STZ (two-way ANOVA, *P* < 0.05; Fig. 5c) compared to Ctrl. Summarized according to the temporal resolution of the other metrics, daily UI significantly increased on day 22 compared to Ctrl (two-way ANOVA, *P* < 0.05; Fig. 5d). However, the UI in 1 day resolution revealed a significant increase in both STZ groups already on day 20 compared to Ctrl (two-way ANOVA, *P* < 0.05; Fig. 5e). From day 29 to 57, the average UI increased 8.6-fold in ‘STZ low’ (4.5 ± 0.6 UI/mouse/day) and 16.6-fold (8.7 ± 0.8 UI/mouse/day) in ‘STZ high’ compared to Ctrl (two-way ANOVA, *P* < 0.05; Fig. 5f). Thus, water intake and UI were the most sensitive indicators of developing hyperglycemia, with UI outperforming manual glucose measurements in STZ groups by 2 and 9 days, respectively. In fact, with the longest latency from treatment to measurable effect in ‘STZ low’, intermittent glucose testing turned out to be the least reliable metric. This is no coincidence considering that glucose measurements only reflect the glycemic status exactly at sampling, that is, usually during the light phase. Comparing circadian urination patterns revealed the limitation of studying hyperglycemic models only in light phase (Fig. 5g–i). Before and during STZ treatment, UI patterns were similar between groups (Fig. 5g,h). However, the circadian profile post-STZ treatment revealed a biphasic pattern, with most of the increased UI occurring in the first 9 h of dark phase, followed by a second bout at the beginning of the light phase (Fig. 5i). Investigating the weekly progression of circadian UI, we found that dark-phase polyuria developed in both STZ groups 1 week after treatment (Supplementary Fig. 3e). In the second week, the separation between STZ high and STZ low emerged at the beginning of the dark phase (Supplementary Fig. 3f). A full separation, including early light-phase polyuria, was established by week 3 post-STZ and remained the subsequent 3 weeks (Supplementary Fig. 3g–j).

### UI reversal by pharmacological hyperglycemia treatment

**Fig. 6:**
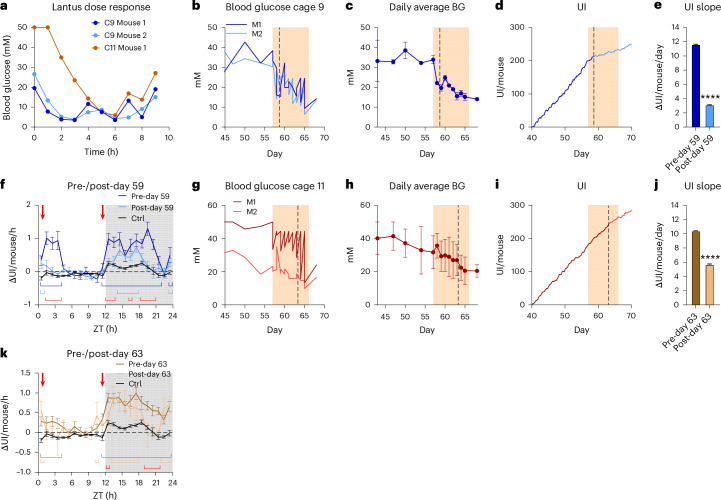
Hyperglycemia intervention for UI reduction. **a**, Blood glucose during the insulin Lantus dose-response test (1 U/mouse, three individual mice). **b**–**k**, Lantus intervention in cage 9 (blue, **b**–**f**) and cage 11 (brown, **g**–**k**). The time course of individual blood glucose (**b**,**g**), daily average blood glucose (BG; mean ± s.e.m.) (**c**,**h**) and UI (**d**,**i**). The shaded area is the treatment period and the dashed line indicates time point of UI slope change. The average daily urination rate before and after the UI slope change (linear regression slope ± s.e.m., ANCOVA with covariate treatment) (**e**,**j**) and the circadian change in UI before and after UI slope change compared to control cages (*n* = 6, repeated-measures two-way ANOVA adjusted with Tukey’s multiple comparisons test) (**f**,**k**). The red arrows show the time of Lantus dosing, brackets in group colors indicate significant differences from Ctrl and red brackets indicate differences between pre and post. *P < 0.05, **P < 0.01, ***P < 0.001 and ****P < 0.0001. Source data

Having established the reliability of the UI for monitoring onset and development of polyuria, we evaluated its applicability to assess the therapeutic efficacy of glucose-lowering intervention with long-acting insulin for mice with constant glucose of >30 mM in weeks 4–6 after STZ treatment (insulin treatment of both mice in cage 9 and one mouse in cage 11). An initial dose-response test to validate effective glucose lowering showed that both cage 9 mice remained around the euglycemic range (blood glucose <10 mM) for 8 h after dosing, while the treated cage 11 mouse took 4 h to reach comparable levels due to the initial >50 mM blood glucose (Fig. 6a). During the subsequent treatment period, glucose, measured at dosing time, was effectively reduced in both cage 9 mice (Fig. 6b) by over 50% (day 57: 33.8 ± 1.4 mM and day 65: 15.2 ± 1.3 mM; Fig. 6c). After 2 days of treatment, the UI trajectory changed substantially (Fig. 6d), corresponding to a 73% reduction in ΔUI (Fig. 6e), and coinciding with the point where glucose reached ~20 mM. This effect was driven by an overall decrease in the circadian urination pattern, with dark-phase polyuria reduced from 11 to 5 h and light-phase polyuria essentially eliminated (Fig. 6f). In cage 11, treatment was also effective but showed higher variability and a distinct pattern of lower afternoon glucose compared to morning (Fig. 6g). During treatment, glucose of both mice gradually declined by 35% (day 57: 31.7 ± 7.0 mM, day 65: 20.5 ± 5.9 mM; Fig. 6h). The UI trajectory changed when average glucose reached ~25 mM (Fig. 6i), corresponding to a 45% reduction in ΔUI (Fig. 6j), which is remarkable given that only one mouse was treated. Again, light-phase urination normalized almost completely to Ctrl, while dark-phase polyuria was blunted and reduced from 12 to 7 h (Fig. 6k).

## Discussion

Our study demonstrates accurate detection of hyperglycemia by continuous bedding monitoring in an automated home cage system, representing a meaningful advance in metabolic research methodology. This approach addresses critical challenges of traditional glucose measurements, which are invasive, time consuming and only intermittent snapshots of glucose levels^16^. The strong correlation between bedding moisture and blood glucose during hyperglycemia validates our metric as a reliable biomarker, particularly in severely diabetic mice, showing up to 8.5-fold increased UI.

The correlation between UI and other metabolic parameters reflects the DM pathophysiology, in which polyuria is accompanied by hyperglycemia, polydipsia and polyphagia^22^. Our multiparameter monitoring approach provides insights into the complex diurnal rhythm of hyperglycemia and its effects on voiding behavior^23,24^. Capturing these patterns, especially during the dark phase, is important given that voiding behavior regulation is known to be altered in DM through various mechanisms, including vasopressin-induced aquaporin-2 upregulation and prostaglandin E2-mediated effects^44,45^. This is particularly useful for monitoring (large) colonies that develop spontaneous hyperglycemia, such as the non-obese diabetic mice, which traditionally require frequent sampling between 12 and 30 weeks of age^46^. This aligns with current efforts to improve continuous glucose monitoring in animal models while reducing sampling stress^4,13^.

The noninvasive UI substantially enhances animal welfare by eliminating stress and stress-induced blood glucose fluctuations associated with traditional sampling methods^11,47^. Handling-free sampling can be done via catheterization^48^ or CGM but this requires surgical expertise, specialized setup and skilled personnel, which either is still limited to glucose snapshots or, for CGM, needs calibration with handling and can affect glucose regulation^17,18^. Our approach circumvents such limitations while providing continuous data. Our findings demonstrate the method’s sensitivity to therapeutic interventions, as evidenced by successfully reversing the polyuria phenotype following pharmacological insulin treatment of hyperglycemic STZ mice. Tracking treatment responses in real time enables precise monitoring of intervention efficacy or timing of treatments according to the onset of hyperglycemia, potentially reducing model variability and improving research outcomes. Although water intake correlates strongly with UI, UI provides substantial advantages by eliminating potential manual errors such as water spillage and by providing important insights into circadian rhythmicity that are not possible through time-restricted, manual volume weighing of consumed water.

A key advantage of our approach is its group-housing compatibility, avoiding alterations associated with individual housing, which is, for example, required in metabolic cage phenotyping. Individual housing has been shown to affect energy intake and expenditure, potentially inducing anxiety- and depression-like behavior that could confound experimental results^20,49^. Monitoring polyuria in group-housed mice in their home cage minimizes the adverse effects of human presence and environmental changes^50^. Conventional methods, including VSAs, require handling and induce stress, capturing only discrete snapshots of voiding behavior. VSAs are artifact prone as some mice do not urinate at all during the experiment, which is not natural. Additionally, the stress caused by removing mice from their home cage can trigger urination immediately before the assay and thus affects test results. Moreover, VSA experiments are usually performed during the light phase, so findings tend to correspond to a disturbed sleep phase. In contrast, our UI-based approach facilitates continuous, hands-free monitoring, substantially reducing stress-related data fluctuations, providing a circadian profile and improving welfare as well as data quality.

Moreover, the DVC simultaneously collects data beyond BSI, such as circadian activity or changes of sleep patterns^39,51^, enabling multifaceted characterization with more complex data compared to blood glucose assessment alone. This might highlight novel aspects or effects of treatments or genotypes. Integrating this methodology with existing DVC technology builds upon established applications in animal husbandry management and welfare monitoring^52,53^. This approach extends beyond traditional void spot assays and metabolic cage analyses, which induce artifacts and stress in laboratory animals^29,30^.

The UI offers clear refinement as it enables continuous, noninvasive monitoring of hyperglycemia development in mouse models and eliminates the need for frequent stressful procedures such as tail vein pricking or surgical CGM implantation. This reduces stress and pain and improves animal welfare. Its compatibility with social housing avoids single housing and isolation-related confounders and home cage monitoring reflects more natural behavior. By enabling longitudinal tracking in the same mice, UI also supports reduction of animal use through improved data resolution, quality and statistical power. While not a full replacement, it minimizes invasive sampling, marking a major step toward more ethical and reproducible metabolic research.

However, several limitations warrant consideration. The susceptibility of the UI to artifacts from water spillage or bottle leakage during cage handling necessitates careful monitoring and documentation of maintenance tasks. Although the DVC uses IVC cages, fluctuations in ambient humidity can affect the bedding moisture measurement and thus affect the accuracy of the UI. Therefore, to ensure the consistency and reliability of UI readings, it is critical to maintain stable environmental conditions within established guidelines (55 ± 10% relative humidity), which can be evaluated by inspection of the readings from the Rack Environmental Monitor module on the DVC. Furthermore, as evaporation characteristics vary with bedding materials and amounts, controlled tests should be performed to identify the optimal bedding amount to minimize evaporation variations. Particularly in models with only mildly increased urination, evaporation can be a serious confounder, especially if bedding is not held constant. Another important consideration for UI tracking when sharing a DVC rack for multiple experiments is cage and position changes, as external, unrecognized activities can affect the target experimental cages. Therefore, all (cage) parameters should be accurately captured and kept consistent during UI recordings, which in turn benefits the data quality. The long-term advantages in terms of data reliability, animal welfare and operational efficiency makes this technology scientifically beneficial for laboratories involved in (metabolic) research and fulfilling important 3R aspects in many in vivo experiments with its continuous cage monitoring, especially in projects with possible moderate or severe burden. Additionally, while the correlation between blood glucose and UI is robust, the detection sensitivity may be reduced in group-housed animals with only mild hyperglycemia, similar to challenges faced when using other glucose measuring systems^21^. Moreover, although we demonstrated the robustness of the UI across various mouse strains and models (CD1, outbreed SWISS, C57BL/6J, ob/ob), inherent genetic- and sex-related metabolic differences may influence urination behaviors. Therefore, future research is warranted to systematically evaluate the UI biomarker across broader mouse populations, including multiple genetic backgrounds, sexes and research environments, to ensure its universal applicability.

The UI excels as a robust biomarker, especially for pronounced hyperglycemia (≥20 mM) with substantial polyuria. In mild hyperglycemia (10–20 mM), where polyuria is less pronounced, it can be detected on a cage basis, but its sensitivity decreases and complementary individual blood glucose measurements are required. The UI cannot properly distinguish between a euglycemic and a hyperglycemic mouse in one cage or two mildly hyperglycemic mice. In addition, averaging UI across group-housed mice may mask individual metabolic differences, which is critical when interpreting data from mixed diabetic populations. Considering this, we suggest an adaptation of the experimental design, that is, monitoring one treated and one control mouse together in the same cage. This would ensure full UI detection capability and the study would benefit from a reduction in social stress and an increase in power as the cage number would equal the statistical number of treated mice regarding UI and the control would be carried out under identical conditions.

We developed the UrinatoR app to substantially enhance reproducibility, to allow broad usability and to corroborate the robustness of the UI. The UrinatoR app is freely available (https://www.tsnscientific.com/urinator) and enables independent verification across various laboratories to strengthen confidence in our proposed methodology.

In conclusion, our innovative approach enables a sample-free assessment of hyperglycemia by deriving the digital biomarker UI from BSI. This provides a noninvasive, continuous method for tracking disease onset and progression as well as treatment efficacy. Detecting responses and effects of treatments and other (therapeutic) interventions renders UI highly relevant, especially for pharmacological studies. Maintaining social housing, combined with simultaneous assessment of multiple parameters, makes it a useful tool for large-scale studies and longitudinal monitoring of disease progression. Our methodology represents a meaningful advance regarding the 3R principles for diabetes research, improving animal welfare and data quality. The UI is not a sole metric to assess hyperglycemia but a supporting metric to reduce invasive measures and to estimate the circadian rhythmicity of blood glucose in mouse models of DM.

## Methods

### Ethical statement

All experiments were performed in accordance with Guidance on the Operation of the Animals (Scientific Procedures) Act 1986 and associated guidelines, EU Directive 2010/63, complied with institutional ethical and ARRIVE guidelines and have been authorized by the responsible national authorities. STZ experiments were approved by Landesamt für Gesundheit und Soziales Berlin (G0104/20) and performed at the Forschungseinrichtung für Experimentelle Medizin at Charité – Universitätsmedizin Berlin. The INSPIRE cohort was carried out in accordance with the French Ministry of Agriculture and Toulouse University ethic committee and approved by the Ministry of Superior Education and Research (APAFIS2019120614331282 and APAFIS2020022409196014). Ob/ob mice for urination observations were ordered on license (2019-15-0201-00073).

### DVC monitoring and husbandry conditions

Mice were predominantly group-housed (acclimatization: 7–14 days), fed ad libitum and maintained under standardized site-specific husbandry conditions with 22 ± 2°C, 55 ± 10% relative humidty, a 12-h light/dark cycle (06:00/18:00) in DVC racks (Tecniplast) for data recording. Cohort-specificities are indicated for CD1, ob/ob (SAFE D30 chow, SAFE Aspen bedding), INSPIRE^43^ and STZ^39^ (SAFE FS 14 bedding). As enrichment, cages were supplied with transparent tunnels, translucent red shelters, bite sticks, nesting material and standardized bedding amounts. The DVC home cage-based monitoring system noninvasively tracks electrode data of each cage in a DVC rack via capacitance sensing technology 24/7 in real time. A sensor board containing 12 electrodes under each cage records changes in the electromagnetic fields once every 0.25 s. For our study, we exported BSI data and processed it to acquire the UI, as described in ‘Results’ (Fig. 1) and the ‘App design and workflow’section.

### In vitro water and diet tests and procedures

In vitro tests and procedures are detailed in Supplementary Methods.

### INSPIRE cohort

Voiding behavior data of the INSPIRE mouse cohort was assessed at ages 6, 12, 18 and 24 months^40,43^. We evaluated the DVC data of 123 cages with male and 87 cages with female outbred SWISS mice, housed 4 mice per cage at start of the aging study, for UI 30 days before voiding tests and compared the 30-day UI change to the total area of urine spots. To account for dropouts, ΔUI and total voiding area were corrected for the actual housing density, whereby both metrics reflect the average urination per mouse in the cage.

### Housing density analysis

As a preliminary assessment of UI linearity, we analyzed historical data from a 30-week-old CD1 stock colony (Crl:CD1(ICR), Charles River) housed in the DVC for data recording. Housing densities were one, two, three, four or five males or females per sex and cage. During the recording (34 days), only routine husbandry procedures were performed.

### Ob/ob mice

As the T2DM model, we analyzed DVC data from two individually housed, 25-week-old, male ob/ob mice (B6.V-Lep^ob^/JRj, Janvier Labs), which were part of an unrelated study. We included their DVC and blood glucose data that were consistently above the reading range of handheld glucometers (>33.3 mM; Contour XT/Next, Bayer), providing a model of severe uncontrolled T2DM. During the recording (23 days), only routine husbandry procedures were performed.

To investigate the time course of phenotype development analogous to these ob/ob mice, we performed a longitudinal study as a urination reference with 10 male ob/ob and 16 male and 16 female C57BL/6JRj WT mice (Janvier Labs) starting at 3 weeks of age. Ob/ob males were individually housed in the DVC to enable quantitative comparison of blood glucose and UI. Glucose from tail vein pricking, body weight and water consumption by manual bottle weighing were recorded three times per week at 10:00. Starting around day 27, one ob/ob mouse (labeled Ob #6) developed a stereotypic behavior of excessive grooming on the nozzle of the bottle, causing water to spill in the cage. The WT reference mice were group housed as four per cage, only recording DVC data without assessing glucose, body weight or water consumption.

### STZ pilot study and STZ main study

To induce polyuria, we used the STZ-mediated T1DM model with C57BL/6J WT females (Charles River) for both studies. In the STZ pilot study, ten 8-week-old females were housed in two cages with two mice and two cages with and three mice in the DVC. At 10 weeks, five mice (two cages) were injected with vehicle Ctrl (buffer: 0.1 M citric acid: 0.1 M Na-citrate 3:2, pH 4.0) and five with STZ (55 mg/kg, freshly dissolved and used within 15 min) on 5 consecutive days for pancreatic islet β cell destruction. Owing to expected polyuria, cages were regularly changed twice a week with monitoring of diet and water intake. Blood glucose was monitored in weeks 0, 4 and 8, followed by killing for organ collection.

For the STZ main study, 28 5-week-old females were pair-housed in the DVC system, and at 7 weeks of age, 12 mice (6 cages) were injected with Ctrl and 16 mice (8 cages) with STZ for 5 consecutive days. Cage changes were standardized twice a week, on Monday and Thursday. On the same days, mice were scored and monitored for body weight, diet and water intake and blood glucose measurements in the morning. After reaching constant hyperglycemia (>30 mM) in weeks 4–6, respective mice were treated by intraperitoneal injection of insulin glargine (1 U, Lantus, Sanofi) twice daily between 06:00 and 07:00 and before start of night phase between 17:00 and 18:00. Both mice from cage #9 and one of cage #11 met these inclusion criteria. The other mouse in cage #11 did not and was therefore not treated. Finally, mice were killed for organ collection.

### Statistical and data analyses

Data processing, graph generation and statistical analyses were performed and generated in Excel (Microsoft), Prism 10 (GraphPad) and the UrinatoR App. All data are presented as the mean ± s.e.m. unless indicated otherwise for cage data or individual data points for correlations.

Data were analyzed with a one-way ANOVA, group-wise comparison by ANOVA, repeated-measures two-way ANOVA adjusted with Tukey’s multiple comparisons test for post hoc analysis, ANCOVA (with covariate and factor) or simple linear regression for correlation and 95% confidence interval (CI) as indicated. Sample size or cage number are included accordingly in each figure legend. All tests were two-sided and statistical significance was set as *P ≤ 0.05, **P ≤ 0.01, ***P ≤ 0.001 and ****P ≤ 0.0001.

### App design and workflow

The UrinatoR app was coded in R (v4.3.1 and shiny v1.9.1), available on https://github.com/Mortendall/UrinatoR, and hosted on https://cbmr-rmpp.shinyapps.io/UrinatoR/. App descriptions and instructions are available on https://www.tsnscientific.com/urinator. Package dependencies and version control were managed with renv (v1.0.7).

The app workflow is as follows: after assigning a CSV separator and local decimal mark, the app preprocesses an uploaded CSV file by standardizing column names and removing summary statistics. Time stamps are converted to local time and groups are inferred based on column name. Next, an event list (csv) only filtering cage INSERTION events is uploaded. Each event is assigned to the closest matching data point using the fuzzyjoin package (v0.1.6). Delta value for each time point *X* is calculated as value(*x*) = rawdata(*X*) − rawdata(*X* + 1), and delta values are excluded in a predetermined window around each event to account for water bottle spillage from cage handling and to exclude cage changes. Once delta values have been calculated and data has been trimmed, the user inputs the number of mice per cage to calculate increases per mouse. Cumulative values are calculated for each cage or mouse. Summary stats are generated and DVC signals can be visualized via Plotly (v4.10.4) as raw data, cumulative values or incremental changes for individual cages or experimental groups. Urination can also be visualized per hour for experimental groups or individual cages. All data can be downloaded as an xlsx file.

### Reporting summary

Further information on research design is available in the Nature Portfolio Reporting Summary linked to this article.

## Online content

Any methods, additional references, Nature Portfolio reporting summaries, source data, extended data, supplementary information, acknowledgements, peer review information; details of author contributions and competing interests; and statements of data and code availability are available at 10.1038/s41684-025-01648-8.

## Supplementary information

Supplementary Information Supplementary Methods and Figs. 1–3.

Reporting Summary

Supplementary Data 1 Statistical source data.

Supplementary Data 2 Statistical source data.

Supplementary Data 3 Statistical source data.

## Source data

Source Data Fig. 1 Statistical source data.

Source Data Fig. 2 Statistical source data.

Source Data Fig. 3 Statistical source data.

Source Data Fig. 4 Statistical source data.

Source Data Fig. 5 Statistical source data.

Source Data Fig. 6 Statistical source data.

## Data Availability

All data analyzed or generated in this study are included in the main text or Supplementary Information. The datasets are available from the corresponding authors upon request. The UrinatoR app is available on https://www.tsnscientific.com/urinator. Source data are provided with this paper.
