# Supplementary material for: Robust noninvasive detection of hyperglycemia in mouse models of metabolic dysregulation using the novel Urination Index biomarker
Source: Lab Anim (NY). 2025 Nov 21;54(12):379–89. doi: 10.1038/s41684-025-01648-8 (PMC12657215; doi:10.1038/s41684-025-01648-8)
Supplement: Supplementary file 2 — Reporting Summary [file 41684_2025_1648_MOESM2_ESM.pdf]

nature portfolio

Corresponding author(s): Sebastian Brachs
Thomas Svava Nielsen
Stefano Gaburro

Last updated by author(s): Sep 26, 2025

Reporting Summary

Nature Portfolio wishes to improve the reproducibility of the work that we publish. This form provides structure for consistency and transparency in reporting. For further information on Nature Portfolio policies, see our Editorial Policies and the Editorial Policy Checklist.

Statistics

For all statistical analyses, confirm that the following items are present in the figure legend, table legend, main text, or Methods section.

n/a	Confirmed	
☐	☒	The exact sample size (*n*) for each experimental group/condition, given as a discrete number and unit of measurement
☐	☒	A statement on whether measurements were taken from distinct samples or whether the same sample was measured repeatedly
☐	☒	The statistical test(s) used AND whether they are one- or two-sided<br>*Only common tests should be described solely by name; describe more complex techniques in the Methods section.*
☐	☒	A description of all covariates tested
☐	☒	A description of any assumptions or corrections, such as tests of normality and adjustment for multiple comparisons
☐	☒	A full description of the statistical parameters including central tendency (e.g. means) or other basic estimates (e.g. regression coefficient) AND variation (e.g. standard deviation) or associated estimates of uncertainty (e.g. confidence intervals)
☐	☒	For null hypothesis testing, the test statistic (e.g. *F*, *t*, *r*) with confidence intervals, effect sizes, degrees of freedom and *P* value noted<br>*Give P values as exact values whenever suitable.*
☒	☐	For Bayesian analysis, information on the choice of priors and Markov chain Monte Carlo settings
☒	☐	For hierarchical and complex designs, identification of the appropriate level for tests and full reporting of outcomes
☐	☒	Estimates of effect sizes (e.g. Cohen's *d*, Pearson's *r*), indicating how they were calculated

Our web collection on statistics for biologists contains articles on many of the points above.

Software and code

Policy information about availability of computer code

Data collection	Bedding Status Index (BSI) data was collected using a Digital Ventilated Cage system (DVC, Tecniplast), software version v6.5.0-1824. BSI data were exported via the DVC Analytics v.4.1 platform (Tecniplast).
Data analysis	The Urination index (UI) was analyzed using the UrinatoR app, which was coded in R (v4.3.1 & shiny v1.9.1), available on https://github.com/Mortendall/UrinatoR, and hosted on https://cbmr-rmpp.shinyapps.io/UrinatoR/. App description and instructions are available on https://tsnscientific.com/UrinatoR. Package dependencies and version control were managed with renv (v1.0.7).

For manuscripts utilizing custom algorithms or software that are central to the research but not yet described in published literature, software must be made available to editors and reviewers. We strongly encourage code deposition in a community repository (e.g. GitHub). See the Nature Portfolio guidelines for submitting code & software for further information.

Data

Policy information about availability of data

All manuscripts must include a data availability statement. This statement should provide the following information, where applicable:
- Accession codes, unique identifiers, or web links for publicly available datasets
- A description of any restrictions on data availability
- For clinical datasets or third party data, please ensure that the statement adheres to our policy

All data analyzed and generated in this study are included in the main text or the supplement. The datasets are available from the corresponding authors upon request.

Field-specific reporting

Please select the one below that is the best fit for your research. If you are not sure, read the appropriate sections before making your selection.

☒ Life sciences ☐ Behavioural & social sciences ☐ Ecological, evolutionary & environmental sciences

For a reference copy of the document with all sections, see nature.com/documents/nr-reporting-summary-flat.pdf

Life sciences study design

All studies must disclose on these points even when the disclosure is negative.

Sample size	Mice in STZ 2.0 were paired-housed for each treatment (CTR or STZ) to evaluate drink and food intake as well as cage bedding data. The size of the sample was chosen mainly based on experience and literature, as females respond to the STZ treatment to varying degrees. Therefore, 8 control and 12 treatment cages were used for the study to enable statistical analysis.
Data exclusions	Data associated with cage handling or bedding changes were shown to contain artefacts and were excluded.
Replication	We applied our analysis to both male and female mice from 3 different strains, across five different studies, and the results obtained with our novel biomarker was consistently reproduced in all studies.
Randomization	Mice were ordered from vendors and randomly allocated to experimental cages considering similar weight distribution. Cages were randomly assigned to CTR or STZ treatment.
Blinding	Blining was useless, as STZ cages were clearly distinguishable by cage pollution and water intake. However, this did not affect the assessment of parameters.

Reporting for specific materials, systems and methods

We require information from authors about some types of materials, experimental systems and methods used in many studies. Here, indicate whether each material, system or method listed is relevant to your study. If you are not sure if a list item applies to your research, read the appropriate section before selecting a response.

Materials & experimental systems

n/a	Involved in the study	
☒	☐	Antibodies
☒	☐	Eukaryotic cell lines
☒	☐	Palaeontology and archaeology
☐	☒	Animals and other organisms
☒	☐	Human research participants
☒	☐	Clinical data
☒	☐	Dual use research of concern

Methods

n/a	Involved in the study	
☒	☐	ChIP-seq
☒	☐	Flow cytometry
☒	☐	MRI-based neuroimaging

Animals and other organisms

Policy information about studies involving animals; ARRIVE guidelines recommended for reporting animal research

Laboratory animals	Mice: C57BL/6J (male & female, age 3-18 weeks), CD1 (Crl:CD1(ICR), male and female, age 30-35 weeks), and ob/ob (B6.V-Lepob/JRj, male, age 3-10 weeks)
Wild animals	The study did not involve wild animals
Field-collected samples	The study did not involve samples collected from the field
Ethics oversight	The studies were approved by the Landesamt für Gesundheit und Soziales Berlin, Germany (G0104/20), the French Ministry of Agriculture and Toulouse University ethic committee, and The Danish Animal Experiments Inspectorate (2019-15-0201-00073)

Note that full information on the approval of the study protocol must also be provided in the manuscript.
